# Enhanced Broad-Spectrum Efficacy of an L2-Based mRNA Vaccine Targeting HPV Types 6, 11, 16, 18, with Cross-Protection Against Multiple Additional High-Risk Types

**DOI:** 10.3390/vaccines12111239

**Published:** 2024-10-30

**Authors:** Kosuke Tsukamoto, Akio Yamashita, Masatoshi Maeki, Manabu Tokeshi, Hirotatsu Imai, Akira Fukao, Toshinobu Fujiwara, Koji Okudera, Nobuhisa Mizuki, Kenji Okuda, Masaru Shimada

**Affiliations:** 1 Department of Ophthalmology and Visual Science, Graduate School of Medicine, Yokohama City University, Yokohama 236-0004, Japan; secganka@yokohama-cu.ac.jp (K.T.);; 2 Department of Investigative Medicine, Graduate School of Medicine, University of the Ryukyus, 207 Uehara, Nishiharacho 903-0215, Japan; 3 Division of Applied Chemistry, Faculty of Engineering, Hokkaido University, Kita 13 Nishi 8, Kita-ku, Sapporo 060-8628, Japan; 4 Department of Biochemistry, Faculty of Pharmacy, Kinki University, Higashiosaka, Osaka 577-8502, Japan; 5 Department of Pathology, Faculty of Medicine, Saitama Medical University, Saitama 350-0495, Japan; 6 Department of Molecular Biodefense Research, Graduate School of Medicine, Yokohama City University, Yokohama 236-0004, Japan

**Keywords:** human papillomavirus vaccine, L2, mRNA, challenge study, animal model

## Abstract

Background: Current L1-based human papillomavirus (HPV) vaccines provide type-specific protection but offer limited cross-protection against non-vaccine HPV types. Therefore, developing a broad-spectrum HPV vaccine is highly desirable. Methods: In this study, we optimized mRNA constructs and developed a multivalent L2-based mRNA vaccine encoding L2 aa 2-130, which includes all known neutralizing epitopes from four prevalent HPV types (HPV-6, -11, -16, and -18). We evaluated its immunogenicity in a mouse model and compared the efficacy of a commercially available mRNA delivery reagent with a custom-synthesized lipid nanoparticle (LNP) formulation. Results: We identified that a construct containing E01 (a 5′-untranslated region) and SL2.7 (a poly(A) polymerase recruitment sequence) significantly increased protein expression. The L2-based mRNA vaccine induced robust and long-lasting humoral immune responses, with significant titers of cross-reactive serum IgG antibodies against L2 epitopes. Notably, the vaccine elicited cross-neutralizing antibodies and conferred cross-protective immunity not only against vaccine-targeted HPV types but also against non-vaccine HPV types, following intravaginal challenge in mice. We also found that LNP delivered mRNA more effectively in vivo. Conclusions: The L2-based mRNA vaccine developed in this study shows significant potential for broad-spectrum protection against multiple HPV types. This approach offers a promising strategy for reducing the global burden of HPV-associated cancers.

## 1. Introduction

Human papillomavirus (HPV) is the most common sexually transmitted infection globally [1] and is responsible for 4.5% of all cancers worldwide, underscoring its significant role in the global cancer burden [2]. HPV, a double-stranded DNA virus, primarily targets epithelial tissues in both the anogenital and oropharyngeal regions [3]. More than 200 distinct HPV types have been identified, demonstrating considerable genetic diversity that is crucial for the virus’s pathogenicity and the onset of related diseases [4,5] (https://www.hpvcenter.se/human_reference_clones/ (accessed on 22 October 2024)). These types are categorized into high-risk and low-risk groups according to their oncogenic potential and capacity to induce cancer [6]. High-risk HPV types, particularly HPV16 and HPV18, account for approximately 70% of cervical cancer cases, with other high-risk types, such as HPV31, HPV33, HPV45, HPV52, and HPV58, also contributing substantially to the global burden of HPV-related cancers. Cervical cancer is the fourth most common cancer and the fourth leading cause of cancer-related deaths among women worldwide [7]. All seven high-risk HPV types are included in the nonavalent HPV vaccine (Gardasil 9), which are HPV16, 18, 31, 33, 45, 52, and 58. Specifically, HPV58, though less prevalent globally, is notably significant in East Asia, including China. In contrast, in the Western world, HPV types 16, 18, 31, 33, 45, and 52 are most prevalent in cervical cancer cases [8,9]. In contrast, low-risk HPV types are generally not associated with cancer development but are responsible for benign conditions such as genital warts and low-grade cervical intraepithelial neoplasia (CIN). HPV6 and HPV11 are the most common low-risk types, and while they do not typically lead to cancer, their ability to cause persistent infections and lesions can result in significant morbidity and healthcare costs.

The HPV genome, which is approximately 8000 base pairs in length, encodes several proteins, including the L1 and L2 capsid proteins, both crucial for the viral life cycle and immune recognition [3]. The L1 gene encodes the major capsid protein, forming the virus’s outer shell and serving as the primary immune target. L1 is highly immunogenic and can self-assemble into virus-like particles (VLPs), which are utilized in current prophylactic HPV vaccines. These L1-based vaccines, such as the quadrivalent Gardasil and nonavalent Gardasil, have demonstrated high efficacy in preventing infections by the HPV types included in the vaccine formulation, particularly the high-risk types HPV16 and HPV18 [10]. Despite their success, L1-based vaccines provide primarily type-specific protection, meaning their effectiveness is largely confined to the vaccine-targeted HPV types [11]. The genetic variability among different HPV types, especially within the L1 gene, presents a challenge for achieving broad protection across all HPV types [12]. As a result, there is a pressing need to explore alternative vaccine strategies that can provide wider protection against a broader range of HPV types [13].

One promising approach for developing such broad-spectrum vaccines involves targeting the L2 gene, which encodes the minor capsid protein of HPV [14]. Unlike the L1 protein, L2 contains highly conserved epitopes, particularly in its N-terminal region, which are shared across multiple HPV types. This makes L2 a compelling target for inducing cross-neutralizing antibodies capable of providing broad protection [15]. Notable examples of conserved neutralizing epitopes include the RG-1 epitope (HPV16 L2 aa 17–36) [16] and the epitope within aa 65–85 [17]. L2-based vaccines could significantly improve the prevention of HPV-related diseases by covering a broader range of HPV types, including non-vaccine types. However, L2 peptides/epitopes naturally have low immunogenicity, and the neutralizing response elicited by a single L2 antigen is often inadequate. To overcome these challenges, several strategies have been developed to enhance the immunogenicity and broaden the protective scope of L2-based vaccines. These include using L2 concatemers combined with potent adjuvants [18], presenting L2 epitopes on VLPs [19], and delivering multimeric L2 epitopes/peptides via monomeric protein scaffolds such as FcγR-targeting scaffolds [20], bacterial thioredoxin (Trx) [21], or flagellin scaffolds [22].

The recent advent of mRNA vaccine technology has transformed the field of vaccinology, offering new possibilities for developing more effective and adaptable vaccines [23]. mRNA vaccines function by encoding genetic instructions for specific antigens, which are then translated into proteins by the host’s cells, eliciting an immune response. This platform offers several advantages, including rapid development and production, the ability to encode multiple antigens, and the capacity to induce both humoral and cellular immune responses [24]. The success of mRNA vaccines during the COVID-19 pandemic has underscored their potential for rapid deployment and high efficacy, making them an attractive option for next-generation HPV vaccines [25].

In this study, we optimized an mRNA vaccine construct and developed an L2-based mRNA vaccine containing all the L2 neutralizing epitopes from four prevalent HPV types. Intramuscular vaccination induced long-term humoral responses, broadly neutralizing antibodies, and cross-protective immunity in a mouse model. Finally, we optimized the delivery system of the mRNA vaccine for clinical applications.

## 2. Materials and Methods

### 2.1. Ethics Statement

All animal experiments were conducted in strict compliance with both Japanese and international guidelines. The procedures were carried out in accordance with protocols approved by the Administrative Panel on Laboratory Animal Care at Yokohama City University. Specifically, this study was approved under protocol number F-A-21-049 by the Ethics Committee of Yokohama City University.

### 2.2. mRNA Production

**Figure 1:**
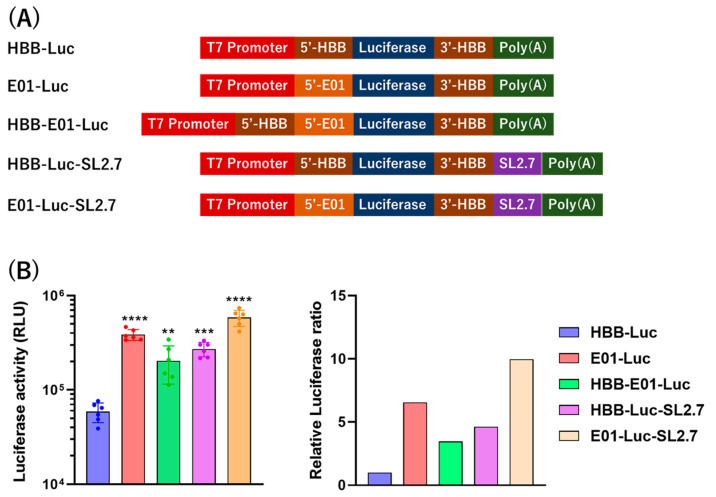
Construction and IVT-mRNA expression in vitro. (**A**) Schematic representation of mRNA constructs. The construct includes the T7 promoter driving the expression of the 5′-HBB (human β-globin) gene and/or E01 (5′-UTR), followed by the luciferase reporter gene, a 3′-HBB sequence, the SL2.7 element, and a poly(A) tail consisting of 114 nucleotides. (**B**) Luciferase activity of constructs in 293TT cells (left panel) and fold change in expression relative to the HBB-Luc construct (right panel). Statistical differences were compared between the HBB-Luc group and other groups. ** *p* < 0.01, *** *p* < 0.001, and **** *p* < 0.0001.

A pBluescript II SK-based plasmid with a T7 promoter site for in vitro mRNA transcription, 5′ and 3′ untranslated regions (UTRs) from human β-globin (HBB), and a 114-nucleotide poly(A) tail, as shown in Figure 1A, was used for mRNA synthesis. Some constructs included the E01 5′-UTR [26] and/or SL2.7, a TENT4 (poly(A) polymerase) recruitment sequence from human cytomegalovirus [27]. Reporter genes (firefly luciferase or NanoLuciferase) or HPV L2 (aa 2-130) from types 6, 11, 16, and 18 were subcloned downstream of the promoter (Figure 1A, and Supplementary Figure S1). The plasmid was amplified in NEB Stable (New England Biolabs, Inc., Tokyo, Japan). To synthesize mRNA, the plasmid DNA was linearized with BSQPI, followed by digestion with proteinase K (100 µg/mL) and 10% SDS. Linearized plasmid DNA was purified using the Wizard SV Gel and PCR Clean-Up System (Promega, Madison, WI, USA). In vitro transcription (IVT) RNA was synthesized using the T7-FlashScribe Transcription Kit (Cellscript, Corp., Madison, WI, USA) according to the manufacturer’s instructions. A 20 µL reaction containing 2 µg of template plasmid DNA was used, in which UTP was replaced with the same concentration of N1-Nethylpseudo-UTP (Jena Bioscience Corp., Jena, Thuringia, Germany) at 37 °C for 30 min. The IVT RNA was capped using the m7G capping system with 2′-O-methyltransferase (Cellscript, Corp., Madison, WI, USA) to produce a cap 1 structure. The synthesized mRNA was then purified using the MEGAclear kit (ThermoFisher Scientific, Corp., Tokyo, Japan).

### 2.3. Drug Delivery System

This study utilized two delivery systems for in vivo mRNA delivery: a liposome-based reagent (Invivojet) and lipid nanoparticle (LNP). For the Invivojet system, mRNA was suspended in mRNA buffer and then mixed with the appropriate amount of in vivo-jet RNA Transfection Reagent (1:1, mg mRNA:mL reagent, Polyplus, Illkirch, France), according to the manufacturer’s instructions.

The LNP preparation method was described in detail in a previously published study [28]. Lipids used in this study were purchased from NOF Corporation (Tokyo, Japan), except for heptadecan-9-yl 8-((2-hydroxyethyl)(6-oxo-6-(undecyloxy)hexyl)amino)octanoate (SM-102) (BLDpharm, Shanghai, China). Briefly, lipids were dissolved in ethanol at molar ratios of 50:10:38:1.5 (SM-102:1,2-distearoyl-sn-glycero-3-phosphocholine (DSPC):cholesterol:1,2-dimyristoyl-rac-glycero-3-methoxypolyethylene glycol-2000 (DMG-PEG)). The lipid mixture was combined with a 10 mM citrate buffer (pH 3.0) containing mRNA at a flow rate ratio of 3:1 (aqueous) using a microfluidic device. The nitrogen-to-phosphate ratio was adjusted to 6. LNP was dialyzed overnight against PBS (pH 7.4) in dialysis cassettes. After the dialysis process, LNP was concentrated using Amicon Ultra centrifugal filters (EMD Millipore, Burlington, MA, USA) and stored at 4 °C until use.

### 2.4. Cells and In Vitro Transfection

293TT cells, derived from 293T cells, with high expression of the SV40 Large T antigen, were kindly provided by Dr. John Schiller’s laboratory (NIH, Bethesda, MD, USA) [29]. The cells were cultured in Dulbecco’s modified Eagle’s medium (DMEM, Wako, Tokyo, Japan) with high glucose, supplemented with 10% fetal bovine serum (FBS) and penicillin–streptomycin (Wako, Tokyo, Japan). mRNA was transfected into 293TT cells using Lipofectamine MessengerMAX (Thermo Fisher Scientific, Tokyo, Japan).

### 2.5. Mouse Model and Immunization

Six-week-old pathogen-free ICR mice were purchased from Japan SLC, Inc. (Shizuoka, Japan). The mice were housed under specific pathogen-free (SPF) conditions in individually ventilated, positive pressure cabinets with controlled temperature, humidity, and a strict 12 h light/dark cycle. Autoclaved bedding was provided, and sterile water and extruded pellet food were available ad libitum. Mice were vaccinated intramuscularly three times with 10 µg of mRNA-L2-Invivojet at 2-week intervals. Licensed Gardasil4, containing HPV-6, -11, -16, and -18 L1 VLPs, was used as a positive control comparator and was dose-normalized to 4 µg HPV16-L1 VLPs per 100 µL injection volume (1/10 of the 40 µg human dose of HPV16-L1 in Gardasil4). Eight and thirty-six weeks after the primary immunization, blood samples were collected for antibody detection. In this study, mRNA vectors were encapsulated in Invivojet, except for the experiments specifically noted as utilizing LNP.

### 2.6. Pseudovirus HPV Preparation

Pseudovirions (PsVs) were prepared as previously described (https://ccrod.cancer.gov/confluence/display/LCOTF/Protocols (accessed on 22 October 2024)). Briefly, humanized L1 and L2 genes from various HPV types (6, 11, 16, 18, 31, 33, 42, 43, 45, 51, 52, 56, 58, and 68) were obtained from Addgene (https://www.addgene.org). The L1 and L2 genes were subcloned into the mammalian expression plasmid (pCAGGS) under the control of the CAG promoter. Plasmids encoding HPV L1 and L2 proteins, along with a reporter plasmid encoding luciferase, NanoLuciferase, or secreted alkaline phosphatase (SEAP), were co-transfected into 293TT cells using Lipofectamine 2000 (Thermo Fisher Scientific Inc., Tokyo, Japan) according to the manufacturer’s instructions. HPV VLPs were purified by iodixanol gradient ultracentrifugation following a previously described protocol [30].

### 2.7. Enzyme-Linked Immunosorbent Assay (ELISA)

ELISA was performed as previously described [31]. Briefly, flat-bottom 96-well MaxiSorp Nunc-Immuno plates (Thermo Fisher Scientific, Tokyo, Japan) were coated with 100 µg/mL of the 19 aa L2 peptide as previously published [32] (for detecting anti-L2 antibodies), or with 1 µg/mL of HPV-16 VLP (for detecting anti-L1 antibodies) in 0.1 M carbonate buffer, and incubated overnight at 4 °C. The plates were then washed 3 times with PBST buffer (PBS with 0.05% Tween-20) and blocked with 0.1% bovine serum albumin (BSA) in PBS at 37 °C for 1 h. The immune sera, diluted 100-fold, was added to the plate and incubated at 37 °C for 1 h.

To quantify HPV-specific serum IgG titers, serial dilutions of isotype mouse IgG in 0.1 M carbonate buffer were coated onto Nunc-Immuno plates overnight at 4 °C, replacing the peptide. After washing the plates 3 times with PBST buffer, an affinity-purified horseradish peroxidase-labeled anti-mouse IgG (GenScript, Tokyo, Japan) secondary antibody was added, and the plates were incubated at 37 °C for an additional 1 h. The plates were developed using o-phenylenediamine dihydrochloride (OPD), and absorbance was measured at 450 nm. A standard curve was generated from the serial dilutions of anti-mouse IgG isotype, plotting the concentration on the *x*-axis (log scale) and absorbance on the *y*-axis (linear). The concentration of the sample was interpolated from this standard curve (https://www.abcam.com/en-su/techinical-resources/protocols/sandwich-elisa (accessed on 22 October 2024)).

### 2.8. In Vitro L2-Pseudovirion-Based Neutralization Assay

The L2-pseudovirion-based neutralization assay was performed as previously described [33,34]. Briefly, cells were cultured in a 96-well plate for 24 h to prepare an extracellular matrix (ECM). The cells were then washed with PBS and lysed with lysis buffer (PBS containing 0.5% (*v*/*v*) Triton X-100 and 20 mM NH_4_OH) at 37 °C for 5 min, followed by 3 washes with PBS. A pseudovirion solution prepared with medium containing recombinant furin and heparin was added to the ECM-coated plate and incubated overnight at 37 °C [35]. The sera were then added to the plate and incubated at 37 °C for 3 h. Afterward, pgsa-745 cells were added and incubated at 37 °C for 2 days. The SEAP activity in the supernatant medium was detected using a SEAP reporter gene assay kit (Roche Life Science Products, Basel, Switzerland). The effective inhibitory concentration (EC_50_) was defined as the serum dilution that inhibited 50% of pseudovirion infection.

### 2.9. In Vivo Bioluminescence Imaging

Mice were intramuscularly injected with luciferase-expressing mRNA (mRNA-Luc). Twelve hours after mRNA administration, the mice were anesthetized in a ventilated anesthesia chamber with 3% isoflurane in oxygen and intraperitoneally injected with 200 µL of D-luciferin (Summit Pharmaceuticals International Corp., Tokyo, Japan) at a concentration of 150 mg/kg. Bioluminescence was observed using the IVIS Spectrum imaging system (PerkinElmer, Waltham, MA, USA). Bioluminescence was quantified in the region of interest to obtain total flux values, expressed in photons per second (p/s), using Living Image 4.0 software provided by PerkinElmer.

### 2.10. Murin Vaginal PsV Challenge

The murine vaginal HPV PsV challenge was performed as previously described, with minor modifications (https://protocolexchange.researchsquare.com/article/nprot-249/v1 (accessed on 22 October 2024)) [29]. Briefly, ICR mice received 3 mg of progesterone (Mochida Pharmaceutical Co., Ltd., Tokyo, Japan) diluted in 100 µL of PBS via subcutaneous injection 4 days before PsV challenge. On the day of the challenge, 40 µL of 5 × 10^4^ IU (infectious units) of PsV (unless otherwise indicated in Supplementary Figure S4) was mixed with 3% carboxymethylcellulose (CMC, volume ratio 1:3). Mice were anesthetized nasally with 2% isoflurane, and an interdental brush was inserted into the mouse vagina, twirling it clockwise and counterclockwise 30 times each. Each mouse was vaginally inoculated with 40 µL of the PsV preparation using an M50 positive-displacement pipette. The mice were then kept in an upward position and incubated for 1 h under anesthesia.

On day 3 post-challenge, the reproductive tract was harvested, dissected, and lysed using the Nano-Glo Luciferase Assay System lysis buffer (Promega, Madison, WI, USA). NanoLuciferase activity in the reproductive tract was measured according to the manufacturer’s instructions.

### 2.11. Statistical Analyses

GraphPad Prism software (version 9.0) was used for statistical analyses and figure preparation. Two groups were compared using a *t*-test (or nonparametric tests, where applicable), and multiple groups were compared using one-way analysis of variance (ANOVA). Degrees of significance were defined as *p* < 0.05 (* *p* < 0.05, ** *p* < 0.01, *** *p* < 0.001, and **** *p* < 0.0001).

### 2.12. AI-Assisted Writing and Language Refinement

In preparing this manuscript, ChatGPT (OpenAI, San Francisco, CA, USA) was utilized for specific language-related tasks, including English language refinement, enhancing sentence structure, and generating draft versions of the Abstract, Introduction, and Discussion sections. All AI-generated content was carefully reviewed and edited by the authors to ensure accuracy, context, and scientific rigor.

## 3. Results

### 3.1. Exploration of IVIS Detection Condition

To determine the optimal time point for IVIS detection, mice were intramuscularly injected with 10 µg of mRNA-Luc encapsulated in Invivojet. Twenty-four hours post-injection, mice were intraperitoneally injected with D-luciferin, and bioluminescence was observed using IVIS every 2 min. Peak bioluminescence activity was detected 10–20 min after D-luciferin injection (Supplementary Figure S2). Based on these results, bioluminescence detection starting 10 min post-D-luciferin injection and lasting for 5 min was used for all subsequent experiments.

To explore the duration of mRNA expression, mice were intramuscularly injected with 10 µg of mRNA-Luc encapsulated in Invivojet. Bioluminescence was detected at 4, 12, 24, 36, and 60 h post-injection. Luciferase expression was detectable at 4 h, peaked at 12 h post-injection, and was undetectable by 60 h post-injection (Supplementary Figure S3). Therefore, bioluminescence was measured 12 h post-mRNA administration in all subsequent experiments.

### 3.2. Optimization of mRNA

A previous study showed that IVT-mRNA containing both the 5′-UTR and 3′-UTR from β-globin generated higher expression levels than those from α-globin [36]. In this study, we generated IVT-mRNA containing both the 5′-UTR and 3′-UTR from β-globin along with four additional constructs to optimize IVT-mRNA expression, as presented in Figure 1A. The IVT-mRNA constructs were transfected into 293TT cells using Lipofectamine MessengerMAX, and luciferase activity in the cells was quantified using a luciferase assay. We found that E01-Luc, HBB-E01-Luc, HBB-Luc-SL2.7, and E01-Luc-SL2.7 induced 6.5-, 3.4-, 4.6-, and 9.9-fold higher expression, respectively, compared to HBB-Luc in the in vitro assay (Figure 1B).

**Figure 2:**
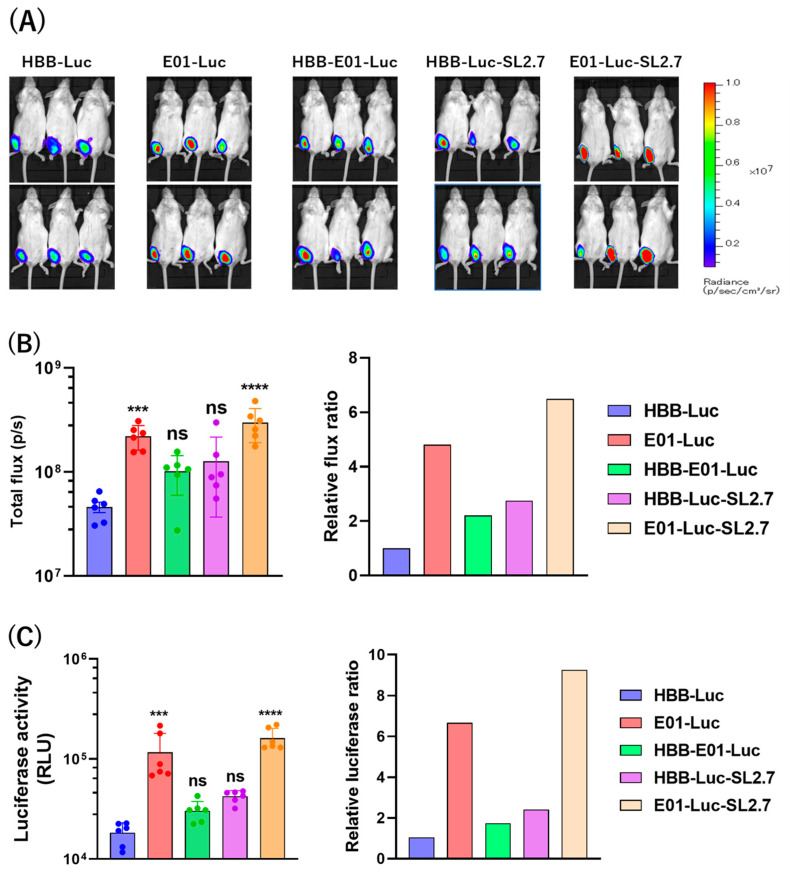
IVT-mRNA expression in vivo. (**A**) A total of 10 μg of mRNA vectors encapsulated in Invivojet was administered intramuscularly to mice, and bioluminescence was imaged using IVIS 12 h post-injection. (**B**) Total flux (p/s) was quantified using Living Image 4.0 software from Perkin Elmer (**left panel**). Fold change in expression relative to the HBB-Luc group (**right panel**). (**C**) Muscle tissue injected with mRNA vectors was removed and lysed using cellular lysis buffer. Luciferase activity was measured in the supernatant of the muscle lysate (**left panel**). Fold change in expression relative to the HBB-Luc group (**right panel**). Statistical differences were compared between the HBB-Luc group and other groups. *** *p* < 0.001, **** *p* < 0.0001, and ns (no significant statistical difference).

Next, 10 µg of each IVT-mRNA construct encapsulated in Invivojet was intramuscularly injected into mice. Twelve hours post-injection, bioluminescence intensity was imaged using IVIS (Figure 2A), quantified with Living Image 4.0 software (Figure 2B), and measured by luciferase assay from muscle tissue (Figure 2C). E01-Luc, HBB-E01-Luc, HBB-Luc-SL2.7, and E01-Luc-SL2.7 induced 4.8-, 2.2-, 2.8-, and 6.5-fold higher expression than HBB-Luc, respectively, as measured by IVIS, and 6.7-, 1.7-, 2.4-, and 9.3-fold higher expression, respectively, by luciferase assay. These results demonstrate that IVT-mRNA constructs containing both E01 and SL2.7 enhance protein expression compared to their parent construct. Therefore, the E01-Luc-SL2.7 construct was used for further study.

### 3.3. Multivalent Prophylactic HPV Vaccine Design

**Figure 3:**
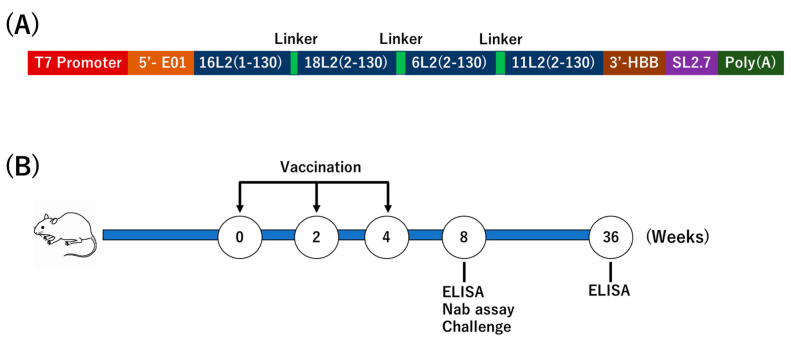
Schematic drawing of mRNA-L2 vaccine and scheme of immunization. (**A**) The mRNA-L2 vaccine construct consists of a T7 promoter, E01 5′-UTR, epitopes encompassing amino acids 2-130 of the L2 protein from HPV-16, -18, -6, and -11, a spacer (GSGGSG), 3′-HBB, the SL2.7 element, and a poly(A) tail. (**B**) ICR mice were intramuscularly vaccinated with 10 μg of mRNA-L2 encapsulated in Invivojet, or Gardasil4, three times at 2-week intervals. Bleeding and assays were performed at weeks 8 and 36.

Compared to the L1 protein, the L2 protein is highly conserved, with all neutralizing epitopes located at its N-terminal region (aa 2-130) [15]. We designed an mRNA vaccine containing L2 aa 2-130 from mucosal oncogenic HPV types 16 and 18, and mucosal low-risk types 6 and 11 (referred to as mRNA-L2) (Figure 3A and Supplementary Figure S1). We aimed to explore whether these four HPV types could induce cross-neutralizing antibodies and cross-protective immunity against other HPV types.

### 3.4. L2 Vaccine Induces Humoral Immunity

**Figure 4:**
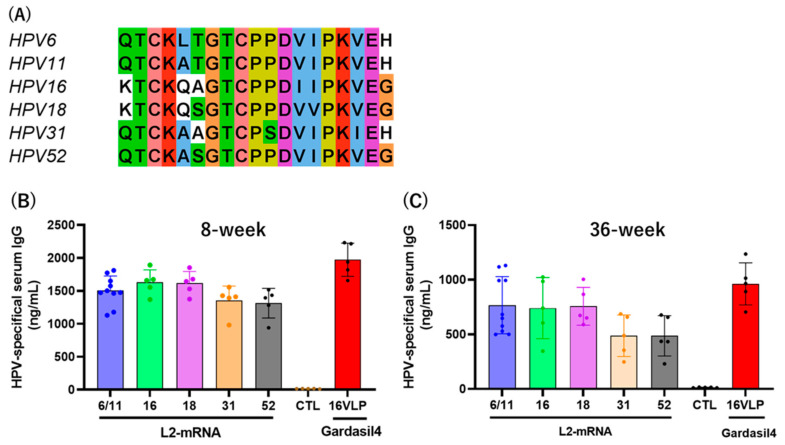
Serum antibody titers. (**A**) Alignment of peptide sequences used for ELISA. Sera were isolated from mice immunized with mRNA-L2 and Gardasil4. Serum antibody titers against specific HPV type peptides were detected by ELISA at weeks 8 (**B**) and 36 (**C**) post-immunization. Each group contained five mice.

Mice were intramuscularly immunized with the mRNA-L2 vaccine or the Gardasil4 vaccine, which contains HPV-6, -11, -16, and -18 L1 VLPs (Figure 3B). Eight and thirty-six weeks after the last immunization, HPV-specific serum antibodies were detected by ELISA using HPV type-specific peptides (Figure 4A). Eight weeks post-immunization, the mRNA-L2 vaccine induced 1504–1630 ng/mL of serum IgG antibodies against HPV-6/11, -16, and -18 L2 epitopes, and 1355 and 1313 ng/mL of antibodies against HPV-31 and HPV-52 L2 epitopes, respectively (Figure 4B). The Gardasil4 vaccine induced 1972 ng/mL of serum IgG antibodies against HPV-16 VLPs. Thirty-six weeks after the last immunization, the mRNA-L2 vaccine induced 741–767 ng/mL of serum IgG antibodies against HPV-6/11, -16, and -18 L2 epitopes, and 487 and 488 ng/mL of antibodies against HPV-31 and HPV-52 L2 epitopes, respectively. The Gardasil4 vaccine induced 962 ng/mL of serum IgG antibodies against HPV-16 VLPs (Figure 4C). The serum IgG titer at 36 weeks was approximately half of that at 8 weeks.

### 3.5. L2 Vaccine Induces Broadly Neutralizing Antibody

**Figure 5:**
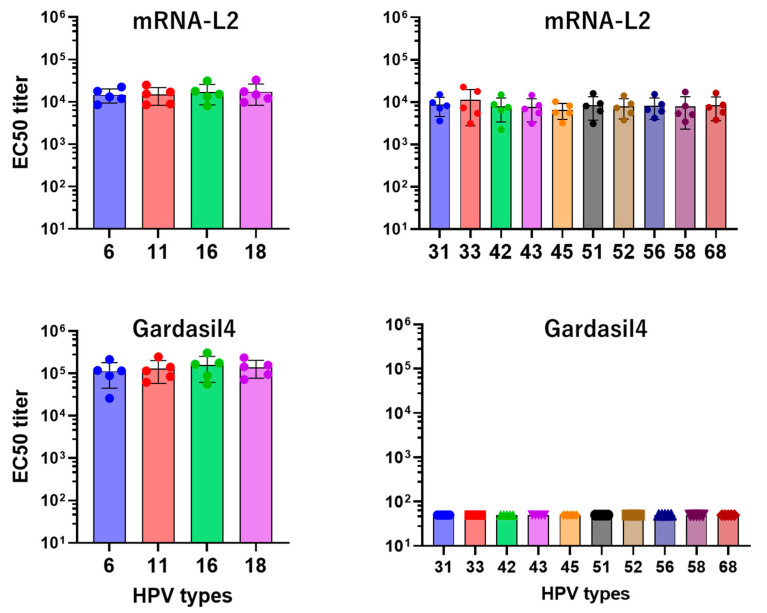
L2-pseudovirion-based neutralizing antibody. Sera were isolated from mice immunized with mRNA-L2 and Gardasil4. The neutralizing antibody titers of mRNA-L2 and Gardasil4 against HPV types were detected using an L2-pseudovirion-based neutralization assay at 8 weeks post-immunization. Each group consisted of five mice.

The mRNA-L2 vaccine induced a 1:14,794–1:17,436 neutralizing antibody titer against HPV-6, -11, -16, and -18, and induced a 1:6602–1:11,322 neutralizing titer against other 10 HPV types. The Gardasil4 vaccine induced a 1:112,500–1:158,280 neutralizing antibody titer against HPV-6, -11, -16, and -18, but neutralizing antibodies against other HPV types was not detectable (Figure 5).

### 3.6. Murin Vaginal Psv Challenge

To explore the sensitivity of the vaginal challenge using HPV PsV carrying NanoLuciferase, 500–500,000 IU of PsV was administered. NanoLuciferase activity positively correlated with PsV dose (Supplementary Figure S4), and NanoLuciferase activity was detectable with as little as 500 IU of HPV PsV in the challenge experiment.

**Figure 6:**
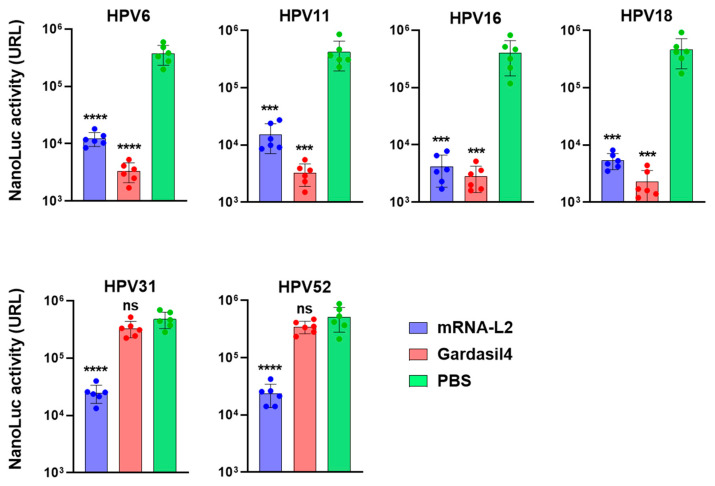
Murine PsV vaginal challenge. Mice were intramuscularly injected with mRNA-L2, Gardasil4, or PBS three times at 2-week intervals. Four weeks after the last immunization, the mice were vaginally challenged with NanoLuciferase-carrying VLPs of HPV-6, -11, -16, -18, -31, or -52. NanoLuciferase activity in the vagina was detected 72 h post-challenge. Each group consisted of six mice. Statistical differences were compared between the HBB-Luc group and other groups. *** *p* < 0.001, **** *p* < 0.0001, and ns (no significant statistical difference).

To evaluate the mRNA-L2 vaccine’s capacity to induce broad protective immunity, mice were immunized with the mRNA-L2 vaccine. Gardasil4 and PBS were used as positive and negative controls, respectively (Figure 6). The HPV PsV challenge led to a 99.1–99.6% reduction in viral load in Gardasil4-vaccinated mice for the vaccine-targeted HPV types, compared to the negative control group. However, for non-vaccine HPV types (HPV-31 and HPV-52), the reduction was only 32.7% and 41.4%, respectively. In contrast, the mRNA-L2 vaccine achieved a 96.4–99.0% reduction for vaccine-targeted HPV types and a 94.8–95.4% reduction for non-vaccine HPV types (HPV-31 and HPV-52). These results demonstrate that the mRNA-L2 vaccine induces strong cross-protective immunity.

### 3.7. Comparison of mRNA Delivery System with Invivojet and LNP

**Figure 7:**
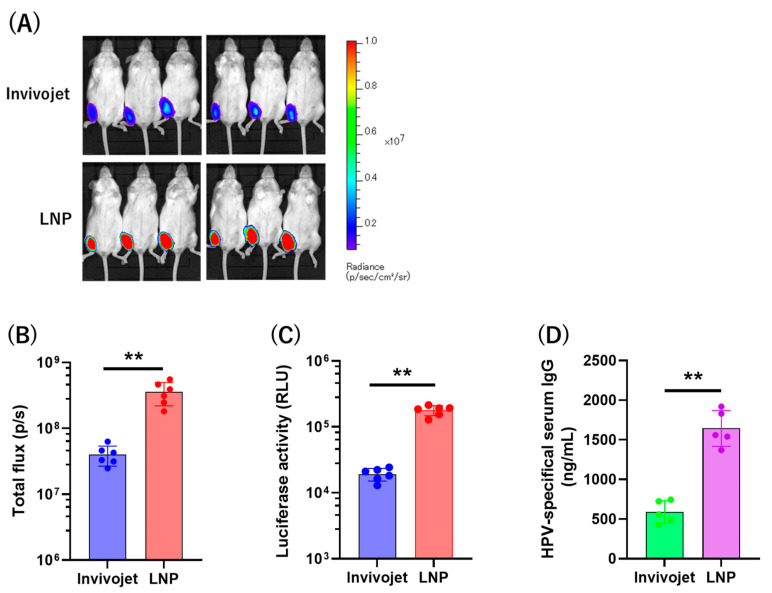
Comparison of Invivojet delivery reagent and lipid nanoparticle (LNP). (**A**) A total of 1 μg of luciferase-expressing mRNA encapsulated iin Invivojet or LNP was intramuscularly injected into mice, and bioluminescence was detected 12 h post-injection using IVIS. Each group consisted of six mice. (**B**) Total flux was quantified, and (**C**) luciferase activity at the injection site in muscle was detected by a luciferase assay. (**D**) The mice were intramuscularly injected with 1 μg of mRNA-L2 encapsulated in Invivojet or LNP three times at 2-week intervals. HPV16-specific serum antibody levels were detected 4 weeks after the last immunization by ELISA. Each group consisted of five mice. Degrees of significance were defined as *p* < 0.05 (** *p* < 0.01).

For clinical use, we compared two mRNA delivery reagents: the commercially available product Invivojet and custom-synthesized LNP, the latter of which has been used for COVID-19 mRNA vaccines. We found that Luciferase protein expression from mRNA-Luc using LNP was 8.91-fold higher than that with Invivojet, as measured by IVIS (Figure 7A,B), and 9.25-fold higher as detected by luciferase assay (Figure 7C). Next, we intramuscularly immunized mice with the mRNA-L2 vaccine encapsulated in either Invivojet or LNP. The vaccine encapsulated in Invivojet induced 589.6 ng/mL of HPV16-specific antibodies, while the vaccine encapsulated in LNP induced 1644 ng/mL (Figure 7D). These data demonstrate that LNP is more effective at delivering mRNA in vivo, resulting in a significantly higher humoral immune response compared to Invivojet.

## 4. Discussion

In this study, we optimized an mRNA construct and generated an L2-based mRNA vaccine that induces cross-protective immunity against multiple HPV types. Notably, we demonstrated that LNP is significantly more effective than the commercial product Invivojet for mRNA delivery in vivo.

Current licensed HPV vaccines, such as Gardasil and Cervarix, have been instrumental in reducing the incidence of HPV-related cancers by targeting the virus’s L1 major capsid protein. Despite their success, these vaccines offer limited cross-protection against non-vaccine HPV types due to their type-specific nature [37]. As a result, the L2 protein of HPV has emerged as a promising target for developing a new generation of vaccines capable of overcoming these limitations [14,19,37]. The L2 protein includes highly conserved epitopes across a broad range of HPV types, particularly within its N-terminal region (aa 2-130), which is incorporated in our designed mRNA-L2 vaccine (Figure 3A and Supplementary Figure S1) [15,38]. L2 peptides and epitopes are inherently poorly immunogenic. To address this challenge, various studies have attempted to present L2 epitopes on bacteria [39], HPV VLPs [40], eukaryotic viruses [41], and bacteriophages [37]. However, the displayed peptides or epitopes are often restricted in length. For a broadly neutralizing antibody response, a vaccine ideally needs to include long L2 peptides containing all neutralizing epitopes from multiple HPV types. In this context, mRNA vaccines present a highly effective approach to HPV vaccine development.

Our study focused on developing and testing an L2-based mRNA vaccine designed to generate broad-spectrum immunity against multiple HPV types. This vaccine encoded L2 epitopes from four prevalent HPV types (HPV 6, 11, 16, and 18). Our results showed that the mRNA-L2 vaccine elicited robust and long-lasting humoral immune responses, producing significant serum IgG titers against both the vaccine-targeted HPV types and non-vaccine types, such as HPV 31 and 52 (Figure 4). Importantly, we observed significant neutralizing antibody titers against the targeted HPV types (6, 11, 16, and 18) and cross-neutralizing antibodies against additional types, such as HPV 31 and 52 (Figure 5). This broad-spectrum immunity aligns with previous research supporting L2 as a vaccine target, as the conserved nature of L2 epitopes promotes the development of cross-protective antibodies capable of neutralizing a wide array of HPV strains [17,18,19]. The L1-based Gardasil4 vaccine induced a strong neutralizing antibody and protective responses against vaccine-targeted HPV types, but it lacked efficacy against non-vaccine HPV types (Figure 5 and Figure 6). In contrast, our L2-based vaccine offers a more universal approach to reducing the global burden of HPV-related diseases by providing protection against both oncogenic and non-oncogenic HPV types (Figure 6).

To enhance vaccine efficacy, the sequence spacer between protein domains plays a crucial role [42]. We employed a flexible GSGGSG linker between the L2 protein domains of each HPV type, ensuring independent folding and function without interference. This linker, composed of glycine and serine, is known for its flexibility and solubility and is commonly used in vaccine vector designs due to its non-immunogenic nature [43,44]. To prevent nucleic acid homology and recombination, we utilized a different codon of this linker for DNA synthesis (Supplementary Figure S1).

Optimizing mRNA constructs is essential for maximizing translational efficiency, stability, and immunogenicity. A critical strategy involves refining the 5′-UTR and 3′-UTR, which regulate mRNA translation and stability [45]. In this study, we used the E01 sequence [26] as the 5′-UTR and a poly(A) polymerase recruit sequence (SL2.7) to extend the poly(A) tail [27], resulting in a significant increase in expression both in vitro and in vivo (Figure 2) [27,46]. While this optimization produced favorable results, further refinements may be necessary to ensure consistent immune responses across diverse populations [47].

Another key consideration is the delivery system for the mRNA vaccine. We compared two methods: a liposome-based reagent (Invivojet) and lipid nanoparticle (LNP) [48]. While both methods effectively delivered the mRNA and induced immune responses, LNP exhibited superior performance in terms of protein expression and immunogenicity. LNP is widely regarded as the preferred mRNA delivery method due to their ability to protect mRNA from degradation, enhance cellular uptake, and improve vaccine stability [38].

In PsV vaginal challenge experiments, most studies have used luciferase-carrying HPV PsV followed by IVIS analysis [29,49]. However, it is time-consuming and requires specialized equipment with limited sensitivity. To address these challenges, we developed a NanoLuciferase-carrying HPV PsV challenge assay, which proved to be highly cost-effective and 100 times more sensitive than the standard luciferase assay. Our challenge experiment succeeded with as little as 500 IU of HPV PsV, a low dose that more closely mimics natural infection (Supplementary Figure S4).

## 5. Conclusions

In this study, we successfully optimized an mRNA construct and developed an L2-based mRNA vaccine that induced cross-protective immunity against multiple HPV types. Additionally, we found that LNP outperforms Invivojet for mRNA delivery in vivo.

## Data Availability

The raw data supporting the conclusions of this article will be made available by the authors without undue reservation.

## Supplementary Materials

The following supporting information can be downloaded at: https://www.mdpi.com/article/10.3390/vaccines12111239/s1, Figure S1: DNA sequence of the expression cassette in mRNA-L2; Figure S2: Sequential detection of bioluminescence activity; Figure S3: mRNA expression progression; Figure S4: Sensitivity of murine vaginal challenge using various doses of HPV PsV carrying NanoLuciferase.
