# Supplementary material for: Enhanced Broad-Spectrum Efficacy of an L2-Based mRNA Vaccine Targeting HPV Types 6, 11, 16, 18, with Cross-Protection Against Multiple Additional High-Risk Types
Source: Vaccines (Basel). 2024 Oct 30;12(11):1239. doi: 10.3390/vaccines12111239 (PMC11598371; doi:10.3390/vaccines12111239)
Supplement: Supplementary file 1 [file vaccines-12-01239-s001.zip › vaccines-3258308-supplementary.pdf]

Supplementary Material list……………………………………………………...2

Figure S1: DNA sequence of the expression cassette in mRNA-L2………………3

Figure S2: Sequential detection of bioluminescence activity………………………3

Figure S3: mRNA expression progression………………………………………..4

Figure S4: Sensitivity of murine vaginal challenge using various doses of HPV PsV carrying NanoLuciferase ………………………………………………………..4

16L2(aa1-130)

ATGCGGCACAAGCGGAGCGCCAAGAGAACAAAGAGAGCCAGCGCCACACAGCTGTACAAGACCTGC
AAACAGGCCGGCACCTGTCCTCCAGACATCATCCCTAAGGTGGAAGGCAAGACAATCGCCGACCAGA
TCCTGCAGTACGGCAGCATGGGCGTGTTCTTTGGCGGACTCGGAATCGGCACAGGCTCTGGAACAG
GTGGCAGAACCGGCTATATCCCTCTGGGCACCAGACCTCCAACCGCCACAGATACACTGGCCCCTGT
TAGACCTCCTCTGACCGTGGATCCTGTGGGCCCTAGCGATCCTAGCATCGTGTCCCTGGTGGAAGAA
ACCAGCTTCATCGATGCCGGCGCTCCTACCTCCGTGCCTTCTATTCCTCCTGATGTGTCT

GSGGSG

GGCAGCGGCGGCTCTGGC

18L2(aa2-130)

GTGTCCCATAGAGCCGCTAGAAGAAAACGGGCCAGCGTGACCGACCTGTACAAAACATGCAAGCAGT
CTGGAACTTGCCCTCCAGACGTGGTGCCTAAGGTTGAGGGAACCACACTGGCCGACAAAATTCTGCA
GTGGTCCAGCCTGGGCATCTTCCTCGGAGGACTTGGCATTGGCACTGGATCTGGTACTGGCGGCAG
GACAGGCTACATTCCTCTCGGCGGCAGATCCAACACCGTGGTGGATGTGGGACCAACCAGACCACCA
GTGGTCATTGAGCCAGTGGGCCCAACAGACCCCTCTATCGTGACCCTGATTGAGGACAGCAGCGTGG
TCACAAGTGGCGCCCCAAGACCAACCTTTACCGGCACCAGCGGATTCGACATCACA

GSGGSG

GGCAGCGGAGGATCCGGC

6L2(aa2-130)

GCCCATTCTAGAGCCAGACGTAGGAAGAGGGCAAGCGCTACCCAACTTTATCAGACTTGCAAACTGA
CCGGGACTTGCCCGCCTGACGTCATACCCAAGGTGGAACACAACACCATTGCAGATCAGATACTGAA
ATGGGGCTCTCTTGGAGTCTTTTTCGGTGGCCTTGGCATCGGCACTGGTAGTGGAACCGGTGGACGT
ACTGGTTACGTGCCCCTCGGAACATCTGCCAAGCCTAGCATTACCAGCGGACCCATGGCTCGTCCTC
CAGTGGTTGTGGAACCAGTCGCTCCCAGCGATCCCAGTATCGTTTCTCTCATTGAAGAGTCTGCCATT
ATCAATGCCGGGGCACCCGAGATCGTGCCACCAGCTCATGGCGGATTCACCATTTAA

GSGGSG

GGAAGTGGCGGCAGCGGA

11L2(aa2-130)

AAGCCTCGGGCCAGACGAAGAAAGAGAGCATCCGCTACACAACTCTATCAAACGTGTAAAGCAACCG
GGACATGCCCTCCGGATGTCATTCCTAAGGTCGAGCACACAACAATTGCTGACCAAATCCTGAAGTG
GGGCAGCCTCGGTGTCTTTTTCGGTGGACTCGGTATTGGCACCGGCGCTGGCAGCGGAGGCAGAGC
AGGATATATTCCTCTGGGCTCTAGCCCCAAGCCTGCCATTACAGGTGGACCTGCTGCTAGACCTCCA
GTGCTGGTTGAACCTGTGGCTCCAAGCGACCCTTCAATCGTTAGCCTCATCGAAGAGAGCGCCATCA
TCAACGCTGGCGCACCTGAAGTCGTGCCTCCAACACAAGGCGGCTTTACCATCACT

Figure S1. DNA sequence of the expression cassette in mRNA-L2.

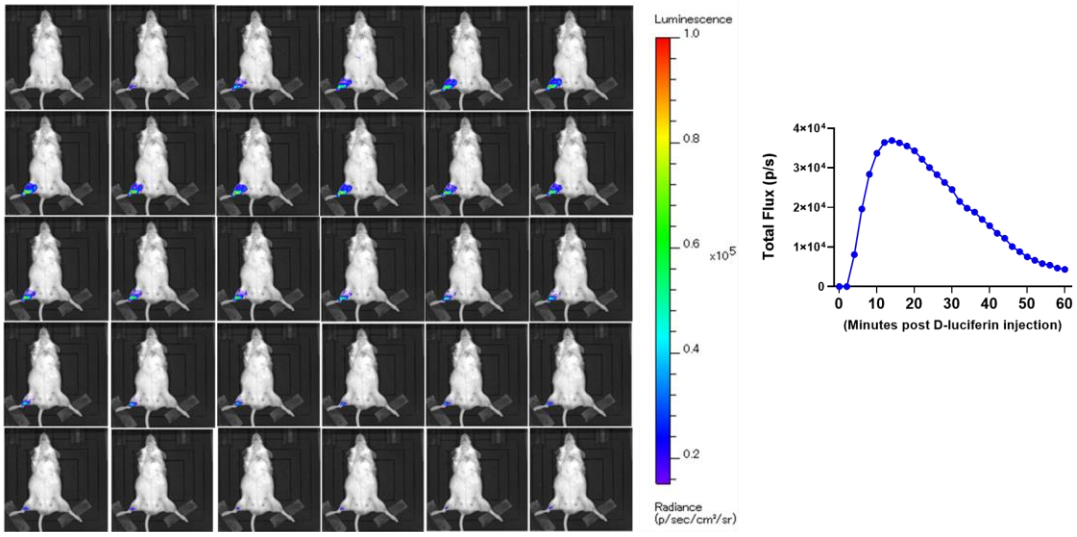

Figure S2. Sequential detection of bioluminescence activity. Ten μg of mRNA-Luc was intramuscularly injected into mice. Twelve hours post-injection, the mice were intraperitoneally injected with D-luciferin, followed by IVIS detection every two minutes.

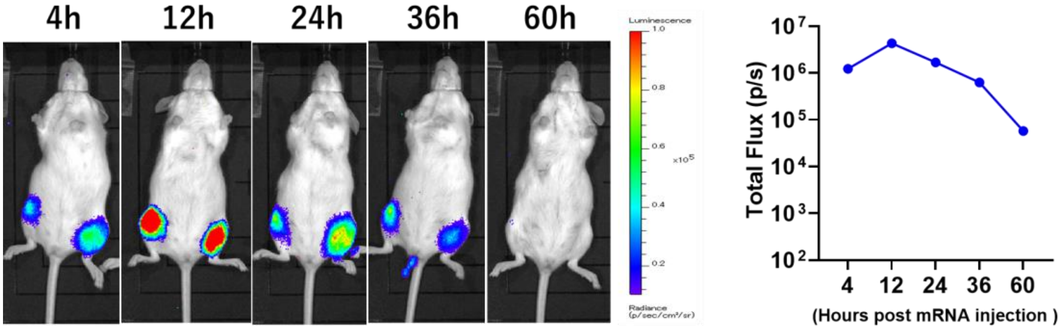

Figure S3. mRNA expression progression. Ten µg of mRNA-Luc encapsulated with Invivojet was intramuscularly injected into mice, and bioluminescence was detected by IVIS at 4, 12, 24, 36, and 60 hours post-injection.

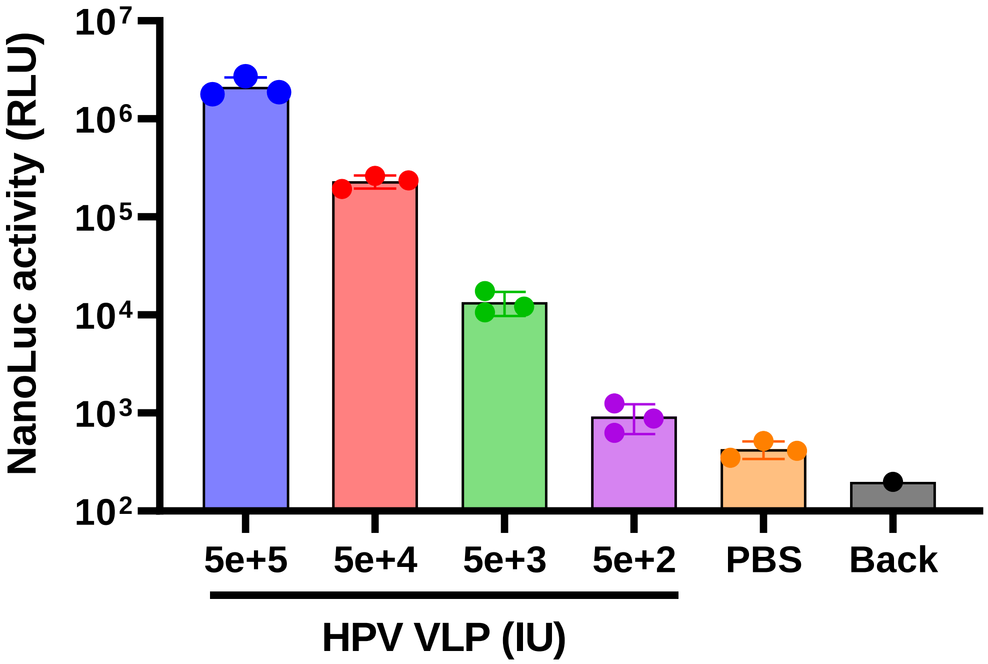

Figure S4. Sensitivity of vaginal challenge using various doses of HPV PsV carrying NanoLuciferase in mice. Mice were vaginally challenged with Nanoluciferase-carrying PsV of HPV-16. NanoLuciferase activity in the vagina was detected 72 hours post-challenge. Each group consists of 3 mice.
